# The Role of Innate Immunity in Conditioning Mosquito Susceptibility to West Nile Virus

**DOI:** 10.3390/v5123142

**Published:** 2013-12-13

**Authors:** Abhishek N. Prasad, Doug. E. Brackney, Gregory D. Ebel

**Affiliations:** Department of Microbiology, Immunology and Pathology, Colorado State University, Fort Collins, CO 80523, USA; E-Mails: Abhi.Prasad@colostate.edu (A.N.P.); Doug.Brackney@colostate.edu (D.E.B.)

**Keywords:** West Nile virus, innate immunity, RNAi, Toll, Imd, Jak/STAT, autophagy, apoptosis, arbovirus, mosquito

## Abstract

Arthropod-borne viruses (arboviruses) represent an emerging threat to human and livestock health globally. In particular, those transmitted by mosquitoes present the greatest challenges to disease control efforts. An understanding of the molecular basis for mosquito innate immunity to arbovirus infection is therefore critical to investigations regarding arbovirus evolution, virus-vector ecology, and mosquito vector competence. In this review, we discuss the current state of understanding regarding mosquito innate immunity to West Nile virus. We draw from the literature with respect to other virus-vector pairings to attempt to draw inferences to gaps in our knowledge about West Nile virus and relevant vectors.

## 1. Introduction

Arthropod-borne viruses (arboviruses) perpetuate in nature in transmission cycles involving both vertebrate animals, including human beings, and hematophagous arthropods, mainly mosquitoes and ticks. The genus *Flavivirus* (family *Flaviviridae*), including dengue viruses 1–4 (DENV), yellow fever virus (YFV), tick-borne encephalitis virus (TBEV), Japanese encephalitis virus (JEV), West Nile virus (WNV) and many others is the most significant group of arboviruses. Several billion individuals, mainly residing in the tropics, are currently at risk of infection with one or more of these agents, and despite the availability of safe vaccines for some of them, several hundred thousand human infections occur annually. The relatively intractable public health burden imposed by arboviruses and the extreme difficulty in designing and implementing effective countermeasures against them stems in part from the complexity of transmission cycles that involve arthropod populations. Mosquito populations, for example, may be influenced by habitat and host availability, temperature and rainfall. Subtle fluctuations in any of these factors may have unpredictably dramatic impacts on the dynamics of virus replication in mosquitoes and human and animal disease [1,2,3,4]. Moreover, mosquitoes are clearly critical to the lifecycle and epidemiology of arboviruses, including WNV, the subject of this review.

Basic studies of the molecular mechanisms that underpin the host-virus interaction in WNV have proceeded vigorously in recent years. These studies, which aim to identify and characterize mechanisms that influence the dynamics of virus infection and contribute to clinical outcomes, have led to a greatly enhanced understanding of how hosts and viruses interact. For example, the importance of vertebrate innate immunity [5,6,7], T-, B-, and other lymphocytes [8,9,10] and complement [11,12,13] have been shown to influence WNV disease to varying degrees. Importantly, the most important vertebrate immune effectors are absent in mosquitoes. Therefore, despite the significance of mosquitoes to WNV perpetuation and transmission, comparatively little is known about the molecular mechanisms that influence the mosquito-WNV interaction. Studies of mosquito responses to WNV (and other arboviruses) are thus in their infancy. 

Work on mosquito-arbovirus interactions has addressed two related points. First, studies have sought to characterize antiviral pathways activated in mosquitoes following virus infection and determine how they limit virus replication (*i.e.*, how do they protect mosquitoes from lethal infection). Second, they have sought to understand how mosquito antivirus responses contribute to the likelihood that a given mosquito will become infected by a virus, support its replication and dissemination beyond the mosquito midgut (the site of initial infection) and ultimately transmit virus (*i.e.*, the mosquito’s “vector competence”). An important question that has not yet been fully explored is the extent to which these two related points (antiviral responses and vector competence) are linked. It has been more or less implicitly presumed that antiviral responses in mosquitoes ought to contribute in some way to the phenotype of vector competence. That this is true of naturally occurring mosquito vectors of arboviruses is far from clear: The literature demonstrates that vector competence is a quantitative genetic trait under the control of several loci that account for a surprisingly small proportion of the observed variation in vector competence [14,15,16]. A full accounting of the functional genomics and systems biology of the mosquito antivirus response is required in order to push forward our current understanding of vector competence. 

**Figure 1:**
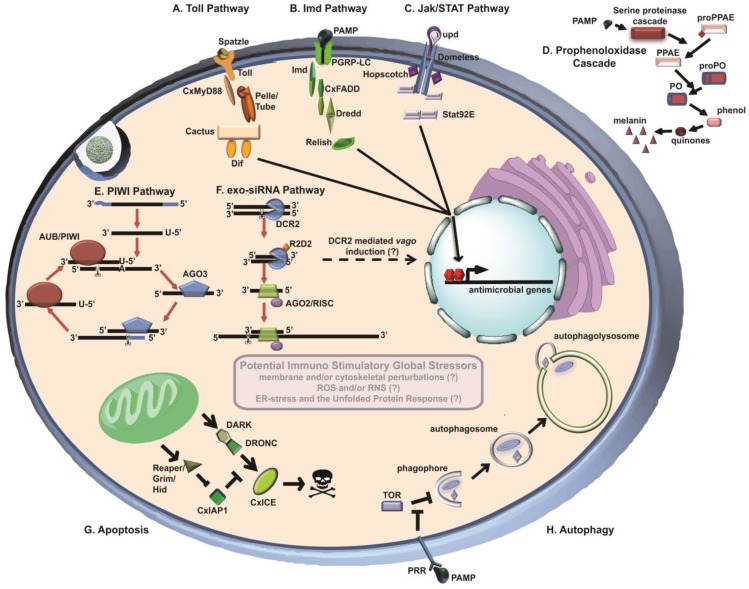
Mosquito innate immune pathways. (**A**) Toll-pathway signaling is initiated by Spӓtzle 5 binding to the Toll receptor, initiating the dissociation of Dif from Cactus. Dif translocates to the nucleus where it initiates transcription of antimicrobial genes; (**B**) Recognition of pathogen-associated molecular patterns (PAMPs) by peptidoglycan recognition protein-LC results in activation and translocation of Relish to the nucleus, and subsequent transcription of antimicrobial genes; (**C**) Extracellular unpaired (upd) binds to the transmembrane receptor Domeless, initiating dimerization of Stat92E, resulting in the subsequent transcription of antimicrobial genes. (**D**) The prophenoloxidase cascade is activated by PAMP activation of the serine proteinase cascade. The pro-prophenoloxidase activating enzyme (PPAE) peptide becomes activated upon cleavage by upstream serine proteinases, resulting in the activation of phenoloxidase. Phenoloxidase converts phenol to quinones, and eventually melanin; (**E**) The piRNA pathway may play a compensatory role to the exo-RNAi pathway during arbovirus infection. Primary piRNAs are fed into the “ping-pong dependent” amplification cycle, whereby cleavage of cognate mRNA leads to production of secondary piRNAs. These in turn bind to complementary transcripts, re-starting the cycle. (**F**) The exo-RNAi pathway is initiated by cellular recognition of exogenous dsRNA. DCR2 cleavage of long dsRNA results in 21nt siRNA duplexes, which are loaded into the RISC. Single-stranded guide strand siRNAs direct RISC to viral genomic RNA by sequence complementarity, leading to cleavage of the virus RNA by AGO2. The DExD/H-domain of DCR2 also leads to induction of the secreted peptide *vago*, activating Jak/STAT and leading to downstream activation of the virus-inducible gene *vir-1*, restricting virus replication. (**G**) Signaling by initiator caspases to effector caspases results in programmed cell death. H) Recognition of PAMPs by unknown PRRs inhibits TOR-mediated negative regulation of phagophore formation, leading to mature autophagosomes, which fuse to lysosomes leading to degradation of the contents of the autophagosomes.

Accordingly, this review addresses the current literature surrounding mosquito antivirus responses, with a particular focus on *Culex* mosquitoes and WNV. Specifically, we cover recent literature on small RNA regulatory pathways (siRNA, piRNA and others), antiviral signaling cascades (Toll, Jak-STAT, *etc*.), and the cellular processes of autophagy and apoptosis. Figure 1 has been included to illustrate each of the pathways discussed in this review. Within this review, we draw heavily on the literature generated in related systems, including *Aedes* mosquitoes and the model organism *Drosophila melanogaster*. This tendency reflects a critical shortcoming in the current state of the field: Very little work on the molecular mechanisms that influence virus infection in mosquitoes has been performed using appropriate virus–vector pairs. Nonetheless, we believe that studies conducted using related systems and model organisms provide critical insights, and the way forward for investigators focusing on arbovirus-mosquito interactions. We conclude with remarks intended to summarize the state of knowledge and identify key areas for future research.

## 2. Small RNA Regulatory Pathways

Small RNA regulatory pathways (SRRPs) are an integral component of endogenous pre- and post-transcriptional gene regulation. Three primary classes of small RNAs exist within metazoans: micro-RNAs, (miRNAs), small-interfering RNAs (siRNAs), and PIWI-interacting RNAs (piRNAs), being distinguished by both the size of the small RNA product, and their biogenesis. Invertebrates lack type I and type III interferon (IFN) responses, which are the main innate immune pathway through which vertebrates respond to virus infection. Rather, in invertebrates, there is ample evidence highlighting the role of SRRPs in antiviral innate immunity. Exogenous RNA interference (exo-RNAi) via the siRNA pathway appears to be the primary small RNA response; however, involvement of the piRNA pathway in antiviral defense has recently been described in both mosquitoes and mosquito cell culture. In this section, we will discuss the role of these pathways and their components in the context of antiviral defense to WNV.

### 2.1. Exo-siRNA Pathway

RNA interference (RNAi) was first described in plants as a mechanism for “post-transcriptional gene silencing” [17], and later, “virus-induced gene silencing” [18], two phenomena which, at the time, were seemingly unrelated. Several years after these initial observations, double-stranded RNA (dsRNA) was found to be the trigger for RNAi in *Caenorhabditis elegans* [19] and *Drosophila melanogaster* [20]. In invertebrates and plants, exo-RNAi is induced by cellular recognition of long dsRNAs as pathogen-associated molecular patterns (PAMPs), which naturally occur as viral genome replication intermediates and genomic RNA secondary structures in the case of RNA viruses, and as convergent transcripts in DNA viruses. These dsRNAs are recognized and cleaved by Dicer-2 (DCR2), a cytoplasmic RNase III enzyme, resulting in 19–23 base pair (bp) fragments (predominately 21 bps) termed siRNAs. siRNA duplexes produced in this manner exhibit 5' monophosphates and 3' hydroxyls, as well as two-nucleotide (nt) overhangs on their 3' termini. These siRNAs are then loaded into the Argonaute-2 (AGO2)-containing RNA-induced silencing complex (RISC) through association with a DCR2/R2D2 heterodimer [21]. After the duplex is unwound a single-stranded RNA known as the guide strand remains associated with the RISC, and is 2'-O methylated by the methyltransferase DmHEN1 [22,23], and the complimentary strand, known as the passenger strand, is discarded. The RISC then recognizes cognate mRNA (in this case, virus genomic RNA) by sequence complementarity with the guide strand. Degradation of the target occurs through the Slicer endonuclease activity of AGO2 [24]. Unlike miRNAs, where mismatches between the guide strand and target are tolerated, even a single mismatch in complementarity between a siRNA and its target can result in diminished or abolished silencing [25,26]. In this way, the siRNA pathway acts as a highly potent antiviral pathway in controlling arbovirus infection.

The role of the siRNA pathway in antiviral defense in arthropods has been the subject of intense investigation in recent years. In *Drosophila*, numerous studies have demonstrated that RNAi inhibits virus replication [27,28,29]. Notably, *Drosophila* with a null mutant DCR2 enzyme exhibit ~70% mortality and dramatically higher virus titers when inoculated with Sindbis virus (SINV, *Togaviridae*), as compared to wild-type controls [28]. In mosquitoes, evidence of the involvement of the siRNA pathway during arbovirus infection has been observed in several virus/arthropod pairings, including o'nyong-nyong virus (ONNV, *Togaviridae*) in *Anopheles gambiae* [30], SINV in *Aedes aegypti* [31], and DENV in *Ae. aegypti* [32]. viRNAs produced in response to WNV had been detected in *Drosophila* S2 cells, but not *Ae. albopictus* C6/36 cells [33] due to a dysfunctional siRNA pathway [34] resulting from a single nucleotide deletion introducing a premature stop codon within the open reading frame (ORF) of DCR-2 [35]. One potential pitfall in interpreting the results of these studies is the utilization of non-natural virus/vector pairings and/or infection routes (*i.e*., intrathoracic inoculation), with the exception of Sanchez-Vargas *et al*. [32]. Brackney *et al*. utilized next-generation sequencing (NGS) to profile the antiviral RNAi response to WNV in its natural vector, *Cx. quinquefasciatus* mosquitoes, following peroral infection, and found viRNAs produced in the midgut of mosquitoes at 7 and 14 days post-infection (dpi) [36]. viRNAs produced in this manner were primarily 21 nts in length (indicative of DCR2 processing), and were asymmetrically distributed along the length of the virus genome.

Given the requirement for high target sequence complementarity in siRNAs, it comes as no surprise that RNAi can drive viral diversity and evolution through the generation of RNAi-escape mutants that differ sufficiently from the master sequence. Viral escape from one or a few transfected siRNAs has been observed in a variety of different systems, including hepatitis C virus (HCV, *Flaviviridae*) [37], human immunodeficiency virus-1 (HIV-1, *Retroviridae*) [26], turnip mosaic virus (TuMV, *Potyviridae*) [38], and poliovirus (PV, *Picornaviridae*) [39]. Based on the observation that WNV population structure was more complex in mosquitoes than in birds [40], it was hypothesized that the mosquito RNAi pathway may serve as a potent selective pressure on the virus to favor generation and maintenance of rare mutants. Indeed, a correlation between nucleotide targeting and increased likelihood for corresponding point mutations has been observed [36], though it is important to note that this observation was associative, and does not demonstrate causation. Taken together, these studies highlight the role this pathway may play in mosquito innate antiviral immunity, and shed light on how it may influence virus diversification and evolution. Moreover, the error rate of the virus replicase complex may serve in part as an evolutionary mechanism for circumventing the mosquito siRNA-based antiviral response through the generation of rare mutants which differ sufficiently from the master sequence. This is circumstantially supported by the observation that a chikungunya virus (CHIKV, *Togaviridae*) mutant expressing a high fidelity RNA-dependent RNA polymerase (RdRP) exhibited lower infection and dissemination titers in mosquitoes as compared to the wild-type virus [41]. However, it should be noted that the molecular basis for this observation was not investigated, and that immunological factors other than RNAi could potentially influence the fitness of a genetically homogenous virus population.

Systemic RNAi, first described in plants and worms, is the process by which the siRNA response spreads beyond the site of initiation into surrounding cells and tissues (see [42] for a review). The mechanism of spread differs between plants and animals, with short-distance transport of siRNAs in plants occurring through plasmodesmal junctions connecting cells, and long-distance transport being mediated by the vascular system. In *C. elegans*, spread of the RNAi signal is mediated by members of the SID family of transmembrane transporters [43,44]. Evidence suggests that this process may occur in dipterans as well, and has broad implications in understanding RNAi-based antiviral immunity in these systems. Studies in flies have revealed a systemic RNAi pathway [45,46], and cell-to-cell spread of viRNAs produced in response to Semliki Forest virus (SFV, *Togaviridae*) infection has been demonstrated in mosquito cell culture [47]. In *C. elegans*, primary viRNAs are amplified in a target dependent manner by the endogenous RdRP RRF-1, which contributes to systemic RNAi spread and subsequent maintenance of long-term silencing [48]. Secondary viRNAs produced in this manner are composed entirely of antisense polarity, and exhibit 5' di- or triphosphates, making them structurally unique, and thus a distinct class of small RNA molecules [49,50]. While an RdRP capable of amplifying viRNAs has not been conclusively identified in dipterans, a recent publication suggests that viral RNA produced during flock house virus (FHV, *Nodaviridae*) in *Drosophila* can be reverse-transcribed into viral cDNAs mediated by the reverse transcriptase activity of endogenous long terminal repeat (LTR)-retrotransposons [51]. Viral cDNA produced in this manner may then be integrated into the host cell genome, or circularized into stable, extrachromasomal DNA which can be efficiently transcribed into dsRNAs that can be fed back into the siRNA pathway, leading to a primed immune response, and allowing for a persistent infection to develop. Additionally, viRNAs produced in response FHV infection in *C. elegans* have been observed to be transgenerationally inherited from mother to offspring in successive generations [52], raising the intriguing possibility that similar mechanisms of amplification and non-Mendelian, extrachromosomal inheritance of small RNAs may exist in mosquitoes as well, though it should be noted that to date, there is a lack of experimental data supporting this. Given the aforementioned importance of the RNAi pathway in mosquito innate immunity to viral infection, inheritance of viRNAs might be expected to influence mosquito vector competence and arbovirus populations in nature.

### 2.2. Vago

Cross-talk between SRRPs and other innate immune pathways is an emerging feature of mosquito antiviral defense against arboviruses. DCR2 belongs to the same family of DExD/H-box helicases as the RIG-I-like receptors, which are involved in the induction of the IFN response in mammalian systems. Deddouche *et al*. reported that the secreted peptide *vago* is induced in *D. melanogaster* in response to *Drosophila* C virus (DCV*, Dicistroviridae*) and SINV infection in the fat bodies of flies, and that induction of *vago* was reliant on the amino terminal DExD/H-box domain of DCR2 [53]. Notably, infection by FHV did not induce *vago* expression, likely due to the FHV encoded viral suppressor of RNAi, B2, which binds dsRNA thereby interfering with downstream signaling by DCR2. While *vago* was shown to control virus infection in this study, the mechanism by which it did so was unclear. Pradakar *et al.* further explored *vago*’s role in antiviral immunity using cultured mosquito cells and WNV [54]. It was found that *vago* was effective in limiting WNV infection in *Cx. quinquefasciatus-*derived Hsu cells, and that induction of *vago* resulted in activation of Jak/STAT signaling, leading to the induction of the STAT-dependent virus inducible gene *vir-1*, thereby restricting WNV replication. Although the identity of the cellular receptor for *vago* is currently not clear, studies published to date suggest that components of the RNAi pathway can have diverse, multifunctional roles in controlling arbovirus infections in mosquitoes.

### 2.3. PIWI-Interacting RNA Pathway

Recent evidence suggests that a second class of small RNAs with distinct biogenesis may also be induced in arthropods following virus infection. The p-element induced wimpy testes (PIWI) class of Argonaute proteins were first discovered in 1997 by Lin *et al*. and shown to be potent regulators of spermatogenesis in *Drosophila* [55]***.*** In 2006, several studies were published indicating that PIWI proteins interact with a unique class of small RNAs, named PIWI-interacting RNAs (piRNAs; the nomenclature at the time was variable, and they were also referred to as repeat-associated small interfering RNAs [rasiRNA], which are now considered a distinct subclass of piRNAs) [56,57,58,59,60]. piRNAs exhibit some unique features that distinguish them from miRNAs and siRNAs. First, piRNAs are considerably larger than most miRNAs or siRNAs, the latter two ranging from 20–23 nts in length, the former 24–30 nts. Secondly, piRNAs are produced from single stranded precursor molecules independently of Dicer processing. Like siRNAs, but not miRNAs, piRNAs are modified by DmHEN1, which results in 2'-O-methylation at the 3' terminus of the RNA [23,61]. Additionally, expression of piRNAs shows tissue specificity, with gonadal tissue being highly enriched for this species and associated proteins. Endogenously transcribed and processed piRNAs have been shown to be important repressors of transposable elements (TEs) in these tissues. However, expression of piRNAs has been detected in somatic tissue as well [62,63].

Biogenesis of piRNAs is proposed to occur through two pathways: the primary pathway and the ping-pong dependent amplification loop. In the primary pathway, piRNAs are processed from single-stranded precursor molecules transcribed from genomic loci (piRNA clusters). In flies, primary piRNAs are associated with PIWI and Aubergine (AUB) [64], and are typically antisense to TEs [59]. Primary piRNAs produced in this manner exhibit a strong bias for a uridine residue at the 5'-terminus of the transcript (U_1_). These primary piRNAs are then fed into the ping-pong dependent amplification cycle, whereby after binding to their target transcript, cleavage occurs 10 nts upstream from the 5' terminus of the primary piRNA [65,66]. Thus, Argonaute 3 (AGO3)-associated secondary piRNAs exhibit an adenine residue in the 10 position (A_10_). Secondary piRNAs subsequently bind complementary transcripts, resulting in cleavage at the A_10_-U basepairing, producing piRNAs corresponding to the sequence of the initial primary piRNA they were derived from, thereby restarting the cycle.

Recently, the piRNA pathway has been implicated in antiviral immunity in invertebrates. A possible hypothesis is that the piRNA pathway can act in a compensatory manner, such as when the siRNA pathway is overburdened or suppressed. Indeed, in *Drosophila*, flies with a null mutant PIWI protein exhibited significantly higher titers of WNV in comparison to wild-type controls [33]. Silencing of AGO3 in *An. gambiae* has been shown to result in increased dissemination of ONNV [30], and dsRNA-knockdown of piRNA-pathway component proteins has been shown to result in increased titers of SFV in *Ae. aegypti*-derived Aag2 cells, with knockdown of PIWI-4 in particular showing considerable effect in this regard [67]. Additionally, sequencing of DENV2-infected *Ae. albopictus* C6/36 cells, which, as previously mentioned, have a dysfunctional siRNA pathway [34,35], revealed a shift from the stereotypically predominant distribution of siRNAs (19–23 nts, no significant strand bias) to products consistent with the piRNA pathway (24–30 nts, predominately positive strand, A_10_ bias) [68]. Wu *et al*. described a population of virus-derived small RNAs in their sequencing of persistently-infected *Drosophila* ovary somatic sheet (OSS) cells that held hallmarks of piRNAs; specifically, being between 24–30 nts in length, exhibiting a strong (95%) strand bias, and a preference for a 5' uracil (though, notably, no bias for an A_10_ was seen) [69]. Similarly, 24–30 nt small RNAs produced in response to DENV2 in *Ae. aegypti* mosquitoes have been found by deep-sequencing [70]. Interestingly, piRNA-like small RNAs sequenced in the latter study exhibited no preference for a 5' uracil, and only a slight bias for an A_10_ residue. 

Recently, piRNA-like small RNAs exhibiting characteristics of ping-pong dependent amplification have been sequenced in mosquitoes and mosquito cells after virus infection. Morazzani *et al*. found that piRNA-like small RNAs are produced in the soma (non-ovarial tissue) of both *Ae. aegypti* and *Ae. albopictus* after infection with CHIKV, and that, unlike endogenously transcribed piRNAs, dsRNA was likely the biogenic precursor for virus-derived piRNAs [35]. Likewise, small RNAs from Aag2 and *Ae. albopictus-*derived U4.4 cells, infected with SINV, shared these same characteristics [71]. In the same study, sequencing data from C6/36 cells infected with La Crosse virus (LACV, *Bunyaviridae*) revealed ping-pong dependent signatures in the piRNA-like viral small RNA population. Similar results from small RNA deep-sequencing of virus infected invertebrate hosts has also been seen in Rift Valley fever virus (RVFV, *Bunyaviridae*) infected mosquito cells [72], as well as Schmallenberg virus (SBV, *Bunyaviridae*)-infected *Culicoides sonorensis-*derived KC cells and Aag2 cells, and blue-tongue virus (BTV, *Reoviridae*)-infected *Culicoides* and mosquito cells [73] (though notably, 24–30 nt small RNAs sequenced from BTV-infected cells did not exhibit signatures of ping-pong dependent amplification). The disparity between different virus/host pairings producing piRNAs either possessing or lacking signatures of ping-pong dependent amplification suggests that the piRNA response may be differentially modulated in response to infection by diverse viruses. While no studies profiling the piRNA response in a *Culex* mosquito/WNV pairing have been published, the existing data in these numerous other infection models suggest that it too may play a significant role in this system.

### 2.4. WNV sfRNA as a Viral Suppressor of RNAi (VSR)

Most arboviruses cause persistent infections within their arthropod vectors. This has led to speculation as to how these viruses maintain infection in the face of a robust RNAi response. In addition to the evolutionary mechanisms (*i.e.*, the viral replicase error rate) described above, many plant and insect-specific viruses have developed molecular mechanisms for subverting the host RNAi response. For example, the previously mentioned FHV encodes a protein, B2, which binds to dsRNA and inhibits the function of DCR2, effectively rendering the siRNA pathway inert [74]. Plasmid-expressed LACV NSs protein has been shown to inhibit IFN and RNAi in mammalian cells and mice [75,76]. However, NSs fails to show any RNAi-suppressive effect in LACV-infected C6/36 cells or in NSs plasmid-transfected U4.4 cells infected with SFV [75]. A recent publication demonstrated that DENV NS4b functions as a VSR in human Huh7 cells via inhibition of dsRNA processing by Dicer [77]. However, whether NS4b behaves similarly in mosquitoes has not been investigated, and to date, no VSR activity has been described for an arbovirus protein during mosquito infection. Thus, it is currently not clear how arboviruses establish and maintain persistent infection of arthropods.

Recently, intriguing evidence has been presented that viral subgenomic RNAs may function as VSRs in some cases. All flaviviruses studied thus far produce a sub-genomic RNA product (sfRNA) from the 3' UTR of the virus genome [78]. In WNV, this RNA comprises the last 525 nts of the virus genome. sfRNA is produced by incomplete degradation of the viral genome by the cellular 5'-to-3' exoribonuclease XRN1, which stalls on the conserved pseudoknot-like structures present at the 5'-terminus of the 3' UTR, resulting in large amounts of sfRNA accumulating within infected cells [79,80]. Recent evidence using fluorescent reporter assays suggests that the WNV sfRNA may act as a VSR by interfering with DCR2 in a concentration-dependent manner [81], though this type of assay alone may be prone to misinterpretation, and should be corroborated by genetic rescue experiments [82]. The mechanism through which sfRNA exerts this effect is unclear, but it is hypothesized that it acts as a decoy substrate for DCR2. Interestingly, sfRNA has also been shown to act in a negative feedback loop by suppressing XRN1 activity due to the enzyme stalling at the 3' UTR [83], illustrating that multiple antiviral pathways can be manipulated by this decay product.

## 3. Immune Signaling Cascades

In addition to RNAi, there are numerous other innate immune pathways responsible for protecting insects from pathogenic organisms. These include the Toll, Immune Deficiency (Imd) and the Janus kinase (Jak)/signal transducer and activator of transcription (STAT) pathways, as well as the phenoloxidase (PO) cascade. Early characterization of these pathways revealed that the Toll pathway was activated upon challenge with gram-positive bacteria and fungi whereas the Imd pathway is responsive to gram-negative bacteria. In each case, signal transduction events initiated upon recognition of PAMPs by pattern recognition receptors (PRRs) results in the transcription of downstream effector molecules, specifically antimicrobial peptides (AMPs). The specific factors responsible for Jak/STAT activation and the downstream effector molecules are not well characterized, but numerous gene products are transcriptionally controlled by this pathway. The PO cascade is integral in wound healing and melanization of pathogenic organisms. Activation of PO results from cuticular damage or PAMP recognition. Traditionally thought to confer protection against bacterial, fungal and parasitic pathogens, recent evidence suggests that these pathways may also play a role in antiviral immunity. The details of these pathways have been extensively reviewed previously [84,85].

### 3.1. Toll Pathway

The Toll pathway was originally described in *Drosophila* as an evolutionarily conserved signaling cascade involved in the establishment of the dorso-ventral axis as well as in many other developmental processes [85,86]. It has since been characterized as having a significant role in innate immunity to gram-positive bacteria and fungi [87,88]. More recently, some studies suggest that the Toll pathway may serve an important role in antiviral immunity. In 2005, Zambon *et al*. observed a significant increase in the expression of the Toll regulated AMPs, Drosomycin and Metchnikowin, upon *Drosophila* X virus (DXV; *Birnaviridae*) challenge of *Drosophila* [89]. It was further shown that flies lacking a functional Toll pathway were significantly more susceptible to DXV challenge. Similarly, it was determined that Toll pathway components and AMPs were significantly up-regulated upon DENV infection of *Ae. aegypti* mosquitoes [90]. Further, suppression of Cactus (a negative regulator of Toll signaling) or MyD88 (a Toll signaling adapter protein) resulted in modest but significant reductions and accumulations of infectious DENV particles in the mosquito midguts, respectively [91]. These findings were confirmed by another study in which Toll pathway components were up-regulated upon DENV infection of *Ae. aegypti* salivary glands [92]. In addition, a modest increase in Dif (a Toll inducible NF-κB transcription factor) was recognized during early stages of SINV infection of *Ae. aegypti* [93]. Together, these data suggest that the Toll pathway plays a role in antiviral immunity in insects; however, others have observed conflicting results [94,95,96]. 

Colpitts *et al.* observed significant reductions in the expression levels of the *Ae. aegypti* ortholog of *Drosophila* Spӓtzle 5 (an upstream signaling peptide of the Toll pathway) upon *Ae. aegypti* infection with three flaviviruses; WNV, yellow fever virus (YFV, *Flaviviridae*) and DENV [94]. In addition, transcript levels of the *Ae. aegypti* ortholog to *Drosophila* Toll was reduced upon YFV infection. These findings challenge those previously published by Xi *et al*. [90]. Many confounding factors may account for this difference, including but not limited to experimental design, mosquito strains, virus strains and/ or environmental conditions. The authors of this study did not directly address these discrepancies, but rather suggested that viral-associated reductions potentially indicate an evolved mechanism by which arboviruses suppress antiviral pathways. However, there is currently no evidence supporting this. Using the SFV—*Aedes* U4.4 cell system, Fragkoudis *et al*. observed a down-regulation of Toll pathway component transcript levels upon infection by SFV. Furthermore, prior activation of the Toll pathway did not seem to adversely affect SFV replication in U4.4 cells [95]. From these studies it is difficult to determine with confidence the contribution of the Toll pathway to antiviral immunity of insects as a whole. However, these findings may indicate that Toll-mediated antiviral activity is specific to each virus-insect pairing. With regards to WNV, little is known about the role of this pathway during infection of insects except for the apparent down-regulation of the Spӓtzle5-like cytokine during infection of *Ae. aegypti* mosquitoes and that none of the canonical Toll pathway genes and/or associated AMPs were significantly altered during WNV infection of *Cx. quinquefasciatus* [96]. Further research will be needed to fully elucidate the possible contributions of Toll pathway mediated antiviral immunity during WNV infection of mosquitoes. 

### 3.2. Immune Deficiency (Imd) Pathway

The Imd pathway is another immune signaling cascade of insects and bears striking similarities to the Tumor Necrosis Factor (TNF) pathway of mammals. It was initially described after the identification of a mutant *Drosophila* line that had significantly decreased levels of numerous AMPs, yet maintained normal levels of another AMP, Drosomycin [87,97,98]. These findings indicated that the expression of AMPs was controlled by two or more regulatory cascades. It was later determined that *imd* mutant flies were highly susceptible to infection with gram-negative bacteria yet maintain resistance to gram-positive bacteria and fungi [85]. Recently, the Imd pathway has been implicated in antiviral immunity in insects. The first indication of the potential significance of the Imd pathway during viral infections was observed in the *Drosophila*, cricket paralysis virus (CrPV; *Dicistroviridae*) model [99]. Somewhat paradoxically, the authors found that while AMPs were not up-regulated during infection with CrPV, suggesting that neither the Imd or Toll pathways were responsive to CrPV infection, mutant flies lacking components of the Imd pathway were more susceptible to viral infection resulting in shortened lifespan and increased viral replication. Together, these data suggest that Imd activation is uncoupled from AMP induction during infection with CrPV, and implies that induction of the Imd pathway results in transcription of several other genes with presently unknown roles in immunity [100]. The role of the Imd pathway in antiviral immunity is further supported by the observations of Avadhanula *et al*. and Huang *et al*. Using a novel SINV replicon transgenic *Drosophila* line, the authors observed increased AMP expression as well as a modest but significant increase in susceptibility of Imd-deficient lines to SINV challenge [101,102]. Additionally, Sigma virus (SIGMAV; *Rhabdoviridae*) infection of *Drosophila* induced the expression of numerous Imd controlled AMPs; however, the significance of these observations on SIGMAV replicative fitness or fly survivorship were not assessed [103]. While there have been several studies implicating the role of Imd during antiviral immunity, there have been just as many suggesting otherwise. The induction of almost all AMPs were observed during DXV infection of *Drosophila*, yet mutant flies lacking a functional Rel (an Imd inducible transcription factor) were no more susceptible to DXV challenge than the controls [89]. Further, a lack of AMP induction was observed during DCV infection of *Drosophila* [53,104]. As with the Toll pathway, it is difficult to discern the significance of the Imd pathway in antiviral immunity based on these studies. This uncertainty is further confounded by the fact that the majority of these viruses do not naturally infect *Drosophila*, with the exception of SIGMAV and DCV. 

These ambiguities in the literature also encompass mosquito-virus interactions. Recently, a cecropin-like peptide presumed to be under the control of the Imd signaling cascade was found to be significantly induced upon DENV infection of *Ae. aegypti* salivary glands [92]. Characterization of this peptide revealed that it could potently inhibit DENV and CHIKV replication in mosquito cell culture. However, the significance of its antiviral effect has not been confirmed with *in vivo* knock-down studies. In addition, other studies have observed either down-regulation or insignificant differences in the expression of Imd-controlled AMPs or pathway components during SFV infection of *Aedes* U4.4 cells or ONNV infection of *An. gambiae* [95,105], although in the former study, decreased SFV replication in Imd and Jak/STAT activated U4.4 cells was observed. The biological relevance of these findings is difficult to determine at this time because functional assays assessing their significance during natural infections of adult mosquitoes have not been performed. Studies investigating the role of Imd during WNV infection of mosquitoes are lacking, and it remains to be seen what if any effect this pathway has on shaping antiviral immunity to WNV in relevant mosquito vectors; however, it was demonstrated that Imd gene transcripts and/or associated AMP transcripts were unaltered during WNV infection of *Cx. quinquefasciatus* [96]. 

### 3.3. Jak/STAT Pathway

The Jak/STAT pathway is an evolutionarily conserved pathway first described for its role in embryonic segmentation in *Drosophila* [106]. Subsequently it was determined that it has an important role in antibacterial defense. It is comprised of the three major components, the receptor Domeless, the Janus Kinase (Jak) Hopscotch, and the transcription factor STAT [106,107,108]. Unlike the Toll and Imd pathways, a well characterized subset of inducible effector AMPs have not been associated with this pathway; however, a handful of inducible genes containing a STAT binding site in their promoters have been identified, with some of these gene products appearing to have antiviral effects.

The initial findings implicating a role of Jak/STAT in antiviral immunity in insects were observed in the *Drosophila*—DCV model. By performing microarray analysis on bacterial, fungal, and DCV challenged flies, the authors identified a subset of gene products that were up-regulated during DCV infection but not during fungal or bacterial challenge [104]. Upon closer inspection the authors identified a gene, *vir-1*, that contained the STAT binding site within its promoter and was strongly induced during DCV and FHV challenge. Furthermore, it was demonstrated that Jak/STAT deficient flies were more susceptible to DCV challenge as determined by increased DCV replication and increased mortality. These results were confirmed by studies performed by Kemp *et al*. who also demonstrated that the Jak/STAT pathway was important in *Drosophila* antiviral immunity to CrPV [109]. Interestingly, when Jak/STAT deficient flies were challenged with five other evolutionarily divergent viruses, including SINV, DXV and FHV, the authors found no effect on the survivorship of the flies. These results indicate that Jak/STAT involvement in antiviral immunity may be specific to each virus–insect pairing and not broadly applicable to all systems. 

A role for Jak/STAT involvement in mosquito immunity to arboviruses has been described. Specifically, components of the Jak/STAT pathway as well as Jak/STAT inducible gene products were found to be up-regulated upon DENV infection of *Ae. aegypti* mosquitoes [90,110]. Included in these findings were two novel Jak/STAT inducible genes termed Dengue Virus Restriction Factors 1 and 2 (DVRF1-2). Further, it was demonstrated that suppression of these two genes resulted in 2.5- and 2.2-fold increases in DENV-2 replication in mosquitoes, respectively [110]. In addition, a recent study demonstrated that WNV induces the expression of *vago* in Hsu cells. Subsequent silencing of *vago* increased WNV titers. The antiviral effect of *vago* expression was determined to arise from downstream *vir-1* activation via *vago*-induced Jak/STAT signaling. Studies in mosquitoes will be needed to validate the *in vivo* role of *vago* in controlling arbovirus infection. As with the Toll and Imd pathways others have observed conflicting data on the role of Jak/STAT pathway in antiviral immunity. In these studies there were no indications of Jak/STAT up-regulation upon infection in four separate virus-vector models (ONNV/*An. gambiae*, SFV/*Aedes* U4.4 cells, WNV-DENV-YFV/*Ae. aegypti,* WNV/*Cx. quinquefasciatus*) [94,95,96,105]. However, as previously mentioned, activation of the Jak/STAT pathway prior to SFV infection resulted in reduced viral replication in U4.4 cells. From these studies it is difficult to accurately assess the importance of the Jak/STAT pathway in antiviral immunity. Future studies assessing the importance of this pathway on arboviral infection, dissemination and transmission rates within mosquitoes will help clarify its significance. In the context of WNV and mosquitoes, this holds true especially considering the systems utilized; however, it is difficult to determine the relevance considering that one study was limited to cell culture and the other in an ancillary vector. Additional research into the role of Jak/STAT in antiviral immunity in primary mosquito vectors is clearly required in order to fully understand its possible influence on mosquito antiviral responses.

### 3.4. Phenoloxidase (PO) Cascade

Among arthropods, the PO cascade is an evolutionarily conserved extracellular pathway responsible for wound healing and melanization of bacterial and parasitic pathogens [84]. This pathway can be induced by cuticular damage or upon recognition of PAMPs. This in turn activates a serine protease cascade ultimately resulting in the activation of the prophenoloxidase activating enzyme (PPAE). Active PPAE cleaves the prophenoloxidase zymogen to produce PO, which catalyzes the conversion of mono- and diphenolic substrates to quinones, which is then converted to melanin [84]. While direct melanization of viruses has not been observed, there is evidence that by-products of the pathway may have antiviral effects. It was demonstrated that plasma from the tobacco budworm has virucidal effects on *Helicoverpa zea* single capsid nucleopolyhedrovirus (HzSNPV; *Baculoviridae*) and chemical inhibition of PO resulted in increased viral titers [111,112]. It was also determined that 5,6-dihydroxyindole, a byproduct of the PO cascade, could almost completely inactivate *Autographa californica* nucleopolyhedrovirus (AcMNPV; *Baculoviridae*) *in vitro* [113]. Similar antiviral activity has been observed in mosquitoes. Tamang *et al*. observed increased SINV titers in *Armigeres subalbatus* mosquitoes after suppression of prophenoloxidase I [114]. Such observations were also found during SFV infection of U4.4 cells. Specifically, recombinant SFV over-expressing an inhibitor of the PO cascade was able to replicate to significantly higher titers than the control virus. It was further determined that inhibition of the PO cascade could decrease the survivorship of SFV infected *Ae. aegypti* [115]. The authors went on to demonstrate that the specific effector molecules involved in the antiviral effects were the pathway intermediate quinones. The specific nature of their antiviral effect remains to be determined. Together, these studies highlight the potential role of the PO cascade in insect immunity to arboviruses. These are the first studies to assess the role of the PO cascade in antiviral immunity in mosquitoes and it will be interesting to see if this antiviral effect is conserved among other virus-vector pairings. Future research into the involvement PO cascade during WNV infection of *Culex spp.* mosquitoes is warranted. 

## 4. Cellular Processes

In addition to RNAi and the PAMP-induced signaling cascades previously discussed, evidence suggests that multi-functional cellular processes can have significant effects on arboviruses in arthropods. Cellular processes, such as apoptosis and autophagy, are important in maintaining homeostasis in multicellular organisms and integral to their development. Autophagy, in effect, is the recycling center of the cell. It serves an important role in removing damaged organelles and protein aggregates which can put undue stress on the cell, ultimately reducing their constituent macromolecules to basic molecular building blocks and sources of energy. In addition, it appears to function as an innate immune defense against numerous prokaryotic and eukaryotic intracellular pathogens [116,117]. Interestingly, the role of autophagy during viral infections is not well defined. In some systems, autophagy functions in an antiviral capacity while in others it can be commandeered and utilized in a pro-viral manner [118]. However, to date, little is known about the role of autophagy during arboviral infections of arthropods with the exception of the studies discussed below, which indicate a role in antiviral immunity. Apoptosis is the process of programmed cell death (PCD) which plays an integral role in eliminating old, injured or defective cells from organisms. This process can be differentiated from necrosis by its ability to control the release of cellular components in apoptotic bodies which can be scavenged by phagocytic cells and thereby diminishing any potential immunologic over-reaction [119]. It has been observed that such an approach to stress-induced cell death could predispose viruses and their associated PAMPs to antigen presenting cells. Not coincidently many viruses have devised mechanisms by which to subvert or avoid apoptosis altogether [120]. In recent years it has become evident that apoptosis may function as an antiviral defense in insects especially with regards to arboviruses. 

### 4.1. Autophagy

Autophagy is an evolutionarily conserved pathway that serves an important role in maintaining cellular homeostasis and cell survival [121]. Induction of autophagy results in the formation of double-phospholipid membrane vesicles termed autophagosomes which sequester the targeted organelles and proteins. Subsequently, the autophagosomes fuse with lysosomes forming autolysosomes which mediate degradation of the contents [122]. During normal growth conditions autophagy maintains cellular homeostasis by degrading unwanted or damaged organelles and protein aggregates. In times of cellular stress, autophagy catabolizes these cellular components thereby generating a pool of energy and macromolecules that maintain crucial cellular functions until favorable growth conditions return [121]. In addition, it appears to play a significant role in antiviral immunity in invertebrates. Shelly *et al*. demonstrated that deletion of autophagy-related genes (Atg’s) increased vesicular stomatitis virus (VSV; *Rhabdoviridae*) replication and decreased *Drosophila* survivorship [123]. It was later demonstrated that pre-formed PAMPs in the context of UV-inactivated virus appear to interact with Toll-7 at the plasma membrane which leads to the activation of autophagy independently of the canonical Toll pathway [124]. These studies were the first to examine the antiviral role of autophagy to an arbovirus in the context of an invertebrate host. Additional studies are required in order to clarify whether these findings can be replicated in natural virus-vector pairings.

### 4.2. Apoptosis

Apoptosis is a conserved mechanism of programmed cell death in multicellular organisms [125] that is critical to a variety of biological processes including embryonic development, maintenance of homeostasis, and lysis of virus-infected cells by cytotoxic T-lymphocyes [119]. In vertebrates, apoptosis may be induced through two known pathways: the extrinsic, or death-receptor pathway, and the intrinsic, or mitochondrial pathway [126], though in dipterans only the intrinsic pathway is known to exist [127].

Many viruses from diverse families encode anti-apoptotic genes, such as inhibitors of apoptosis (IAPs), p53-binding proteins, and bcl-2 homologs. This has led to the hypothesis that apoptosis functions as an innate immune pathway in response to viral infection [120]. FHV strongly induces apoptotic events in *Drosophila* DL-1 cells through depletion of the *Drosophila* inhibitor-of-apoptosis-1 (DIAP1), a negative regulator of initiator caspase DRONC and effector caspase DrICE; however replication of FHV is not negatively impacted by apoptosis [128]. Similar depletion of host cell IAPs were observed in DL-1 cells [129] and *Spodoptera frugiperda* (order Lepidoptera) SF21 cells [129,130] infected with AcMNPV, which encodes its own viral IAP, p35, with depletion of IAPs in both cell lines being triggered by viral DNA replication [129]. However, p35-deficient mutant viruses demonstrated reduced infectivity of SF21 cells, and reduced infectivity and lethality in *Spodoptera frugiperda* larvae [131]. Furthermore, studies in flies infected with FHV or AcMNPV reveal that rapid induction of apoptosis by the upstream regulator p53 limits viral gene expression and proliferation [132], further highlighting the importance of apoptosis in controlling virus infection in these systems.

Numerous studies have investigated the role apoptosis plays in arbovirus infection models. Apoptosis is a natural consequence of arbovirus infection in mammalian cells, where cytopathic effect (CPE) is frequently seen; conversely, most arboviruses are thought to cause minimal cytopathology in insect cells, instead resulting in non-lytic, persistent infections [133,134]. However, cell death consistent with apoptosis has been observed in mosquito midgut and salivary gland tissues following infection with a variety of arboviruses [135,136,137,138,139,140,141,142], including WNV. With regards to WNV, apoptosis in the midgut epithelium [138] or salivary glands [136,142] of *Cx. pipiens* or *Cx. quinquefasciatus* mosquitoes was associated with a resistance to infection or reduced ability to transmit virus, respectively. Thus, an attractive hypothesis is that apoptosis functions in an antiviral capacity to arbovirus infection in mosquitoes, and that it may be more likely to occur in mosquitoes incapable of vectoring a particular virus rather than permissive hosts [143,144]. Indeed, pro-apoptotic genes in a refractory strain of *Ae. aegypti* mosquitoes were significantly up-regulated between 24 and 48 h post-infection (hpi) in response to DENV2 infection as compared to a susceptible strain of *Ae. aegypti* or blood-only fed mosquitoes from the same strain [145]. In the same study, dsRNA-knockdown of the caspase inhibitor AeIAP1 in two susceptible strains converted the phenotype from susceptible to refractory, and knockdown of initiator-caspase *AeDronc* in the refractory strain resulted in increased permissiveness to DENV2 infection. The same group previously reported up-regulation of pro-apoptotic genes in the DENV2-susceptible strain in comparison to the refractory strain at 48 hpi [146], indicating that apoptotic events controlling virus infection may be induced acutely and rapidly limit the course of infection. However, it is important to note that neither of these studies directly measured apoptotic events in midgut tissue after DENV2 infection, and that the methods used to measure differential gene expression were different between studies (qPCR and suppressive subtractive hybridization, respectively [145,146]). Conversely, modulation of apoptosis by RNAi-mediated silencing of either apoptosis inhibitors or initiators in *Ae. Aegypti,* subsequently infected with SINV, produced results contrary to the hypothesis that apoptosis acts in an antiviral manner to arbovirus infection in mosquitoes [147]. Specifically, inhibition of apoptosis led to decreased infection and dissemination rates, and induction of apoptosis led to greater infection and dissemination. The authors speculate that experimental systemic induction or inhibition of apoptosis prior to virus infection, in contrast to apoptosis being stimulated in individual cells in response to virus infection, resulting in widespread destruction of structural barriers to viral infection/dissemination, may account for this apparent discrepancy. In summary, the variety of studies in mosquitoes investigating the role of apoptosis in response to arbovirus infection strongly implicate the pathway as contributing to the acute antiviral response; and that either the presence or lack of an effective apoptotic response has important consequences for vector competence in mosquitoes. 

## 5. Conclusions

To date, little work has been done elucidating the contributions of each of the aforementioned pathways with regards to WNV infection of mosquitoes. However, based on the evolutionary relatedness of the virus/invertebrate systems analyzed, informed conclusions can be made with regards to the mosquito’s innate immune response to WNV. Specifically, it is evident from these studies that the exo-siRNA pathway most likely contributes significantly to suppression of WNV replication in mosquitoes and their survivorship. Numerous studies in *Drosophila* and mosquitoes have demonstrated the importance of the exo-siRNA response in innate antiviral immunity in invertebrates [27,28,29] and work with *Culex* mosquitoes indicates that the exo-siRNA pathway actively targets the WNV genome [36], but the influence of this pathway on vector competence remains unclear. The extent to which other RNAi pathways, specifically the piRNA and systemic RNAi pathways, contribute to mosquito innate anti-WNV immunity and vector competence remains to be determined. The findings from such studies will be integral to fully elucidating the contributions of RNAi in mosquito antiviral immunity.

The involvement of other immune pathways, specifically the immune signaling cascades (e.g., Jak/STAT, Imd, Toll, PO) and cellular processes (e.g., autophagy and apoptosis), in mosquito anti-WNV immunity and vector competence is difficult to quantify at this time. While some evidence suggests that the Jak/STAT and Imd pathways and apoptosis may function to limit WNV infection of mosquitoes, their relevance is somewhat diminished because the studies were limited to either cell culture systems or are predicated on observational correlations. Furthermore, the evidence implicating their involvement is highly inconsistent. For instance, while there were no indications of Jak/STAT induction during WNV infection of *Ae. aegypti* in one study, others observed anti-WNV activity associated with the induction of Jak/STAT by *vago* during WNV infection of Hsu cells [54,94]. It has been demonstrated that these observed inconsistencies may result from the inherent variability between virus–invertebrate pairings [109]. Evidence supporting the role of these non-RNAi based immune pathways in antiviral immunity has been described in other arbovirus–mosquito pairings. However, in addition to the inter-study inconsistencies, many of these studies observed that most of these pathways exert marginal antiviral activity. For example, the silencing of components of the Jak/STAT pathway in *Ae. Aegypti,* resulted in an approximately half a log increase in DENV titers [110]. The significance of these findings is not in doubt; however, the question remains: Are these non-RNAi-based immune pathways biologically relevant in invertebrate antiviral immunity and/or vector competence? We would assume that if these pathways were biologically relevant that we would be able to identify evolved signatures of antiviral immune recognition and downstream effector molecules. It is conventionally thought that some if not all of these pathways are induced upon recognition of PAMPs by PRRs. For both RNAi and viral induced autophagy this paradigm has been validated in invertebrates [27,124]; however, no known viral-specific PRRs and/or viral-derived PAMPs have been identified for the other pathways. It could be that such PRRs and/or PAMPs exist, but have yet to be characterized. In addition, only a couple of downstream antiviral effector molecules have been described and their mechanisms of action have yet to be determined [92,104,110]. It will be important that future studies focus on elucidating the signatures of viral immune recognition and the mode of action of downstream effector molecules in order to fully appreciate their involvement in antiviral immunity. 

The possibility remains that such PRR/PAMP interactions are not involved in the induction of these pathways during viral infection of invertebrates. There is an increasing body of literature suggesting that other global stress factors may be controlling the induction of these pathways. Viruses and hosts are locked in an evolutionary arms race with hosts having evolved numerous innate sensing mechanisms and viruses having evolved countermeasures to either diminish their functionality (e.g., VSRs) or ways of evading them all together (e.g., genome sequestration or dsRNA degradation) [74,148,149]. While these countermeasures can be very effective, often times they are very specialized and are unable to conceal broader insults associated with the lifecycle of viruses (e.g., membrane perturbations associated with viral entry). In mammalian cells, several studies have demonstrated that such insults can lead to the induction of interferon stimulated genes independent of interferon production [150,151,152]. Further, perturbations in cytoskeletal integrity and functionality have been shown to induce innate immune responses in mammalian cells [153,154]. During viral infections, the cytoskeleton functions not only as physical barrier, but can be important during viral entry and in transporting viral components throughout the cell [155]. This viral-induced stress could inadvertently induce a global, non-descript innate immune response [156]. In addition, many viruses induce the production of reactive oxygen species (ROS) which have been linked to innate immune signaling pathways [157,158,159]. In fact, oxidative stress has been observed in C6/36 *Ae. albopictus* cells during DENV infection and in *Drosophila* during SINV and FHV infections [109,160]. In addition, Pan *et al*. demonstrated that the Toll pathway can be activated in *Ae. aegypti* upon ROS induction during a *Wolbachia* infection, illustrating the link between the induction of global stress signals and downstream immune signaling cascades [161]. Interestingly, it was observed that *Wolbachia* infections can reduce WNV replication in *Cx. quinquefasciatus* [162]. Further, viruses have been shown to impart endoplasmic reticulum (ER) stress on infected cells which has been associated with antiviral signaling. ER-stress is a broad term that encompasses multiple stress response pathways, all of which converge upon the unfolded protein response (UPR) which subsequently result in immune activation and can often times result in apoptosis [163,164]. Interestingly, it appears that the UPR is up-regulated during DENV infection of mosquito cells and numerous studies have evaluated this response to arboviruses in mammalian cells [165,166,167,168,169]. Currently, it is unknown whether or not these pathways, individually or synergistically, contribute to and account for the discrepancies concerning the antiviral nature of many of these pathways. Therefore, it will be important that future studies examining these pathways consider the possibility that non-canonical activation of immune pathways and/or non-specific global stressors could be responsible for the induction and/or activity of these pathways.

We will conclude by reiterating the statement we made in the introductory paragraph: while a continually expanding body of knowledge is developing regarding mosquito innate immunity to arbovirus infection, there are still major gaps in our knowledge regarding the relevance of these pathways to virus–vector ecology and natural transmission dynamics. While well-established models such as *Drosophila* have been greatly useful due to being easily amenable to genetic manipulation, it is important that relevant virus–vector pairings be investigated to draw sound conclusions that can be extrapolated to the natural world. This effort will be aided with the recent completion of the genome sequences for the three most medically relevant mosquito species, *Aedes aegypti*, *Anopheles gambiae*, and *Culex quinquefasciatus* [170,171,172].
